# Chemical diversity and biological activities of marine sponges from the genus *Tedania*: a comprehensive review

**DOI:** 10.3389/fphar.2026.1763267

**Published:** 2026-04-22

**Authors:** Geane Gabriele Oliveira Souza, José Walber Gonçalves Castro, Lariza Leisla Leandro Nascimento, Ana Maria Fernandes Duarte, Fázia Fernandes Galvão Rodrigues, José Galberto Martins Costa

**Affiliations:** 1 Postgraduate Program Biological Chemistry, Department of Biological Chemistry, Regional University of Cariri - URCA, Crato, CE, Brazil; 2 Natural Products Research Laboratory, Department of Biological Chemistry, Regional University of Cariri - URCA, Crato, CE, Brazil

**Keywords:** alkaloids, antiproliferative, bioactive, lipids, Tedania

## Abstract

Given current public health concerns and the growing number of individuals affected by cancer, inflammatory diseases, and microbial infections, the identification of natural bioactive metabolites has become crucial as a therapeutic alternative. Marine sponges stand out in this context due to their ability to produce diverse bioactive metabolites. The genus *Tedania* aligns with this perspective, as literature reports associate it with cytotoxic, anti-inflammatory, and antimicrobial properties. This integrative review aimed to compile studies on the chemical and biological composition of the genus *Tedania*, correlating molecular structure with biological activity while updating existing knowledge. The databases ScienceDirect, PubMed, LILACS, and Web of Science were searched using the keyword “*Tedania”*. Inclusion criteria comprised articles reporting the identification or isolation of metabolites and/or biological activities, while duplicate and unrelated articles were excluded. A total of 135 compounds were identified for the genus *Tedania*, with lipids predominating (55.55%), followed by alkaloids (21.48%), carotenoids (8.15%), and terpenes (4.44%). Metabolites **20**, **21**, and **22** were detected in three species of the genus. Most of the identified metabolites have not been biologically evaluated; however, alkaloids stood out among those that have been analyzed. Certain substances, fractions, and extracts were tested against various cancer cell lines and pathogenic microorganisms, including assessments of larvicidal activity and key signaling pathways. Regarding the biotechnological potential of the genus *Tedania*, the literature reports three patents with pharmacological applications. Therefore, sponges of the genus *Tedania* represent promising sources of bioactive metabolites with antiproliferative, antimicrobial, larvicidal, and anti-inflammatory effects, which warranting further scientific investigation.

## 1 Introduction

Marine organisms are valuable natural sources for drug development due to their richness in chemical compounds with unique and largely unexplored structures. In addition to exhibiting high metabolic diversity and low toxicity, they represent promising models for the development of bioactive drugs, benefiting various health-related fields (Costa-Lotufo et al., 2009; Shikov et al., 2020).

Among the examples of therapeutic applications are trabectedin, midostaurin, eribulin mesylate, brentuximab vedotin, plitidepsin, enfortumab vedotin, and cytarabine, all of which are used in cancer treatments (Bocharova et al., 2021). Additionally, ziconotide a potent intrathecal analgesic indicated for severe chronic pain (Costa-Lotufo et al., 2009), and vidarabine, used in antiviral therapy (Souza et al., 2025) are noteworthy. It is also important to note that several other compounds are currently at different stages of research and clinical trials (Costa-Lotufo et al., 2009; Bocharova et al., 2021).

Among marine organisms, sponges stand out not only for their bioactive metabolites but also for being structurally simple, sessile, and filter-feeding animals that have existed since the Precambrian period. With the advancement of biological research, poriferans have been employed in the development of scafolding for tissue engineering applications based on stem cells (Tsurkan et al., 2020). They have also been used in environmental applications, such as bioindicators of metal contamination (Marzuki et al., 2020; Vidyalakshmi et al., 2024), biosorbents (Karatepe and Soylak, 2014), bioremediators (Amato et al., 2023), antifouling agents (Acevedo et al., 2013) and bioaccumulators of metallic isotopes (Patel et al., 1985).

The genus *Tedania* (class Demospongiae) has gained prominence due to the pharmacological potential of its secondary metabolites, which have been extensively investigated for their biological activities, particularly cytotoxic (Lhullier et al., 2020; Berne et al., 2015), anti-inflammatory (Costantino et al., 2009; Costantino et al., 2012; Jin et al., 2021), antimicrobial (Quan et al., 2019; Parra et al., 2018), larvicidal (Wantah et al., 2018) and antivenom activities (Faioli et al., 2013). Although the genus comprises approximately 70 sponge species (Hajdu et al., 2011), only nine have been reported in chemical and pharmacological studies over the past 48 years, in addition to seven unidentified specimens. These findings highlight a significant knowledge gap regarding the genus and underscore the need for further studies to deepen the chemical characterization and elucidate the mechanisms of action of its bioactive compounds, which hold potential for therapeutic applications.

**FIGURE 1:**
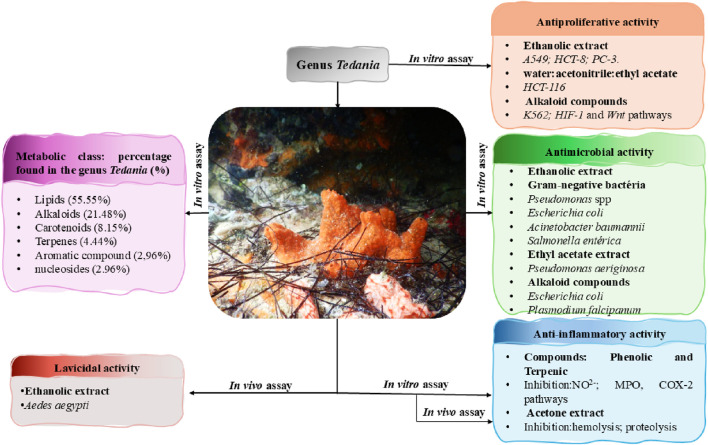
Illustration of the correlation between chemical metabolites from the genus Tedania and their biological activities.

Accordingly, this review compiles information on the chemical composition and biological activities of species within the genus *Tedania* (Figure 1), aiming to support future research efforts by correlating biological effects with chemical structures. By representing the first comprehensive approach to the chemical composition and biological activity of this genus, the present investigation constitutes an initial milestone in the study of *Tedania* marine sponges, providing an up-to-date foundation for advancing chemical, biological, and pharmacological knowledge.

## 2 Materials and methods

**FIGURE 2:**
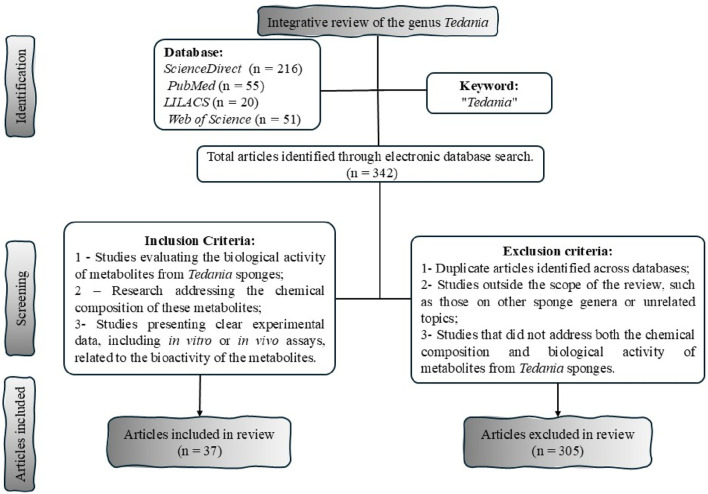
PRISMA flowchart prepared using PowerPoint the software, illustrating the process of identification, screening, and selection of the studies included in the review.

This work was based on scientific studies that addressing the secondary metabolites of marine sponges of the genus *Tedania*, extracted from solvents of different polarities, as well the evaluation of their therapeutic potential. No restrictions regarding time, geographic location, or language were applied. Information regarding the data collection process is exposed in the flowchart below (Figure 2).

This work compiled research conducted on sponges from around the world, China (Zhang et al., 2020; Guo et al., 2024), South Africa (Kibungu et al., 2021), Brazil (Lhullier et al., 2020; Parra et al., 2018), Croatia (De Rosa et al., 2006), Bahamas (Costantino et al., 2009; Costantino et al., 2012), Australia (Quan et al., 2019; Quiann et al., 1980), Russia (Rod′kina, 2005), Caribbean (Dillman and Cardellina, 1991a; Dillman and Cardellina, 1991b), India (Parameswaran et al., 1997), Florida (Schmidtz et al., 1983), Japan (Tanaka and Katayama, 1979), Antarctica (Berne et al., 2015), Venice (Aiello et al., 1993), and Philippines (Banias and Uy, 2023). Samples were collected during different seasons and at various depths, reflecting a wide diversity of environmental exposure.

Although finding studies specifically related to the genus *Tedania* can be challenging, the selected research highlights the importance of chemical and biological investigations, because of the diversity of secondary metabolites and the demonstrated bioactive potential.

## 3 Results and discussion

### 3.1 Metabolites in marine sponges of the genus *Tedania*

The genus *Tedania* belongs to the Family Tedaniidae and the class Demospongiae, comprising approximately 70 described species of marine sponges. Among these, five species have recorded occurrence in Brazil. Among them, *T. ignis* and *T. brasiliensis* are found mainly in the Northeast, Southeast, and South regions of the country, as well as in oceanic áreas of the Atlantic Ocean. *T. ignis* presentes na irregular struture, a slightly rough surface, and red-orange coloration (Hajdu et al., 2011). In contrast, *T. brasiliensis* is characterized by an intense red-pink coloration, occurring preferentially in well-lit environments (Parra, 2016).

Other species of the genus present distinct distribution and morphological characteristics. *T. anhelans* is an encrusting species, capable of attaching to both vertical and horizontal substrates, although it shows greater coverage on vertical surfaces (Knott et al., 2006). In turn, *T. dirrhaphi* is described as a sponges with an irregular, massive-lobed structure, with a smooth surface, whose coloration varies from yellow to golden-yellow, being recorded mainly in the North Pacific Ocean (Stone et al., 2011).

In contrast, no detailed records were found in the consulted literature regarding the taxonomy and zoogeographic distribution of the species *T. digitata* and *T. excavata*, indicating the need for additional studies for better characterization of these species within the genus.

Several factors may influence the metabolic composition of marine sponges, including substrate environment, exposure to contaminants, predation, feeding type, and depth (Batista et al., 2018). This study aimed to compile and discuss the compounds that have been isolated and identified from species of the genus *Tedania*. Extracts and fractions obtained from sponge biomass were prepared using various solvents (both organic and inorganic), followed by processes of isolation, purification, and/or metabolites identification. Experimental procedures involved the use of multiple techniques and analytical methods, including chromatographic (HPLC, UHPLC, LC-MS, etc), spectroscopic (UV, IR, ^1^H and ^13^C NMR 1D and 2D), spectrometric (MS), chemical calculations, and other techniques.

These analyses led to the identification of 135 compounds belonging to various chemical classes, such as: fatty acids (48 metabolites) (Rod′kina, 2005), alkaloids (29 metabolites) (Guo et al., 2024; Dillman and Cardellina, 1991a), sterol (27 metabolites) (Aiello et al., 1993; Montaño et al., 2009; Seldes et al., 1988), carotenoids (11 metabolites) (Tanaka et al., 2001; Uenojo and Maróstica, 2007; Tanaka et al., 1977b; Tanaka et al., 1977a), terpenes (6 metabolites) (Costantino et al., 2009), nucleosides (4 metabolites) (Quiann et al., 1980), aromatic compound (4 metabolites) (Dillman and Cardellina, 1991b), heterocyclic carboxylic acid (2 metabolites) (Parameswaran et al., 1997), macrolide (1 metabolites) (Schmitz et al., 1984), lactam (1 metabolites) (Cronan and Cardellina, 1994), carbazole (1 metabolites) (Parameswaran et al., 1997), and azepine (1 metabolites) (Quan et al., 2019). It is not yet possible to clearly determine which compounds are characteristic of the genus, and further studies are required. However, Table 1 highlights the metabolites reported in more than one species of the genus.

**TABLE 1:** Secondary metabolites reported in more than one species of the genus *Tedania*.

Metabolites	*T. ignis*	*T. anhelans*	*T. excavata*
Cholesta-5,22-dien-3β-ol (19)	+	-	+
Cholest-5-en-3β–ol (20)*	+	+	+
24ξ-Methyl-cholest-5-en-3β-ol (21)*	+	+	+
24ξ-Ethyl-cholest-5-en-3β-ol (22)*	+	+	+
24ξ-Methyl-cholest-5,22(E)-dien-3β-ol (39)	-	+	+
24-Methyl-cholesta-5,24(28)-dien-3β-ol (41)	-	+	+
24ξ-Ethyl-cholesta-5,22-dien-3β-ol (42)	-	+	+
24ξ-Methyl-cholesta-5,22-dien-3β-ol (43)	-	+	+

Legend: (+) presence of the metabolite; (−) absence of the metabolite; (*) metabolite reported in three species of the genus.

These findings contribute significantly to the chemical knowledge base and are valuable to researchers interested in conducting studies the genus *Tedania*. In the following sections, the chemical characteristics of the isolated metabolites are organized by species to facilitate comprehension. Some isolated metabolites, fractions, and extracts were investigated for their cytotoxic, anti-inflammatory, antimicrobial, and larvicidal potential, as presented in Tables 2 and 3.

**TABLE 2:** Isolated metabolites and investigated biological activities in the genus *Tedania*.

Marine sponge	Metabolic class	Investigated biological activity	References
*T. ignis*			
Tedarene A (1)	Diarylheptanoid	Anti-inflammatory	Costantino et al. (2012)
Tedarene B (2)	Diarylheptanoid	Anti-inflammatory	Costantino et al. (2012)
Tedanol (3)	Diterpenoid	Anti-inflammatory	Costantino et al. (2009)
2-Phenylacetamide (4)	Aromatic amide	—	Dillman and Cardellina (1991b)
4-(2′,3′,4′-Trimetyl-phenyl)-but-(3*E*)-en-2-one (5)	Aromatic compound	—	Dillman and Cardellina (1991b)
1-Methyl-carbazole (6)	Carbazoline alkaloids	​	Dillman and Cardellina (1991b)
1-Acetyl-*β*-carboline (7)	Carbazoline alkaloids	​	Dillman and Cardellina (1991b)
3-(Hydroxylacetyl)-indole (8)	Indole alkaloids	—	Dillman and Cardellina (1991b)
4-(Indol-3-yl)-but-(3*E*)-en-2-one (9)	Indole alkaloids	—	Dillman and Cardellina (1991b)
5-(Indole-3-yl)-pent-(3*E*)-en-2-one (10)	Indole alkaloids	—	Dillman and Cardellina (1991b)
6-(Indol-3-yl)-5-methylhepta-(3*E*,5*E*)-dien-2-one (11)	Indole alkaloids	—	Dillman and Cardellina (1991b)
Cyclo(_L_-Pro-_L_-thioPro) (12)	Diketopiperazine alkaloid	—	Dillman and Cardellina (1991a)
Cyclo(_L_-Pro-_L_-Leu) (13)	Diketopiperazine alkaloid	—	Schmidtz et al. (1983)
Cyclo-(_L_-Pro-_L_-Val) (14)	Diketopiperazine alkaloid	—	Schmidtz et al. (1983)
Cyclo(_L_-Pro-_L_-Ala) (15)	Diketopiperazine alkaloid	—	Schmidtz et al. (1983)
Atisane-3*β*,16*α*-diol (16)	Diterpenoid atisane	—	Schmidtz et al. (1983)
Epiloliolide (17)	Terpene lactone	—	Schmidtz et al. (1983)
Tedanolide (18)	Macrolide	—	Schmitz et al. (1984)
Cholesta-5,22-dien-3*β*-ol (19)	Sterol	—	Montaño et al. (2009)
Cholest-5-en-3*β*-ol (20)	Sterol	—	Montaño et al. (2009)
5*α*-Cholestan-3*β*-ol (21)	Sterol	—	Montaño et al. (2009)
24*ξ*-Ethyl-cholest-5-en-3*β*-ol (22)	Sterol	—	Montaño et al. (2009)
24*ξ*-Ethyl-cholest-5,22-dien-3*β*-ol (23)	Sterol	—	Montaño et al. (2009)
23 *ξ*-Methyl-cholesta-5,22-dien-3*β*-ol (24)	Sterol	—	Montaño et al. (2009)
5α-Cholestan-3*β*-ol (25)	Sterol	—	Montaño et al. (2009)
Tedanalactam (26)	Lactam	—	Cronan and Cardellina (1994)
*T. digitata*			
Clathriaxanthin (27)	Xanthophyll	—	Tanaka et al. (2001)
Isoclathriaxanthin (28)	Xanthophyll	—	Tanaka et al. (2001)
Diatoxanthin (29)	Xanthophyll	—	Saxegaard et al. (1981)
Alloxanthin (30)	Xanthophyll	—	Saxegaard et al. (1981)
Isotedaniaxanthin (31)	Xanthophyll	—	Saxegaard et al. (1981), Tanaka and Katayama (1980)
Tedaniaxanthin (32)	Xanthophyll	—	Tanaka and Katayama (1979), Tanaka et al. (1977b), Saxegaard et al. (1981), Tanaka et al. (1978)
Allopurpurin (33)	Xanthophyll	—	Saxegaard et al. (1981)
3-Methoxy-7,8-didehydro-*β,x*-carotene (34)	Xanthophyll	—	Tanaka and Inoue (1988)
Tedanin (35)	Xanthophyll	—	Okukado (1975), Tanaka et al. (1978)
3-Hydroxy-7,8-dihydro-k,*x*-carotene-6,8-dione (36)	Ketocarotenoid	—	Tanaka et al. (1977a)
3,8-Dihydro-k,*x*-carotene-6-dione (37)	Ketocarotenoid	—	Tanaka et al. (1977a)
1-Methylisoguanosine (38)	Nucleoside	—	Quiann et al. (1980)
*T. anhelans*			
Cholest-5-en-3*β*-ol (20)	Sterol	—	Aiello et al. (1993)
5*α*-Cholestan-3*β*-ol (21)	Sterol	—	Aiello et al. (1993)
24*ξ*-Ethyl-cholest-5-en-3*β*-ol (22)	Sterol	—	Aiello et al. (1993)
24 ξ -Methyl-cholesta-5,22(*E*)-dien-3*β*-ol (39)	Sterol	—	Aiello et al. (1993)
Cholest-5,22-dien-3*β*-ol (40)	Sterol	—	Aiello et al. (1993)
24-Methyl-cholesta-5,24(28)-dien-3*β*-ol (41)	Sterol	—	Aiello et al. (1993)
24*ξ*-Ethyl-cholesta-5,22(*E*)-dien-3*β*-ol (42)	Sterol	—	Aiello et al. (1993)
24*ξ*-Methyl-cholesta-5,22-dien-3*β*-ol (43)	Sterol	—	Aiello et al. (1993)
24*ξ*-Methyl-3*β*-cholestanol (44)	Sterol	—	Aiello et al. (1993)
24*ξ*-Ethyl-3*β*-cholestanol (45)	Sterol	—	Aiello et al. (1993)
*β*-Carboline (46)	Carbazole	—	Parameswaran et al. (1997)
Pyrazole-3(5)-carboxylic acid (47)	Heterocyclic carboxylic acid	—	Parameswaran et al. (1997)
4-Methylpyrazole-3(5)-carboxylic acid (48)	Heterocyclic carboxylic acid	—	Parameswaran et al. (1997)
(±) Spondomine (49)	Alkaloid	Cytotoxic, antibacterial, and anti-inflammatory	Jin et al. (2021)
*T. brasiliensis*			
Pseudoceratidine (50)	Bromopyrrole alkaloid	Cytotoxic, and antiprotozoal	Parra et al. (2018)
3-Debromopseudoceratidine (51)	Bromopyrrole alkaloid	Cytotoxic, and antiprotozoal	Parra et al. (2018)
20-Debromopseudoceratidine (52)	Bromopyrrole alkaloid	Cytotoxic, and antiprotozoal	Parra et al. (2018)
4-Bromopseudoceratidine (53)	Bromopyrrole alkaloid	—	Parra et al. (2018)
19-Bromopseudoceratidine (54)	Bromopyrrole alkaloid	—	Parra et al. (2018)
4,19-Dibromopseudoceratidine (55)	Bromopyrrole alkaloid	—	Parra et al. (2018)
Tedamide A (56)	Bromopyrrole alkaloid	Antiprotozoal	Parra et al. (2018)
Tedamide C (57)	Bromopyrrole alkaloid	Antiprotozoal	Parra et al. (2018)
Tedamide B (58)	Bromopyrrole alkaloid	Antiprotozoal	Parra et al. (2018)
Tedamide D (59)	Bromopyrrole alkaloid	—	Parra et al. (2018)
*T. excavata*			
Cholesta-5,22-dien-3*β*-ol (19)	Sterol	​	Seldes et al. (1988)
Cholest-5-en-3*β*-ol (20)	Sterol	—	Seldes et al. (1988)
5*α*-Cholestan-3*β*-ol (21)	Sterol	—	Seldes et al. (1988)
24*ξ*-Ethyl-cholesta-5-en-3*β*-ol (22)	Sterol	—	Seldes et al. (1988)
24ξ-Methyl-cholesta-5,22(*E*)-dien-3*β*-ol (39)	Sterol	—	Seldes et al. (1988)
24-Methyl-cholesta-5,24(28)-dien-3*β*-ol (41)	Sterol	—	Seldes et al. (1988)
24*ξ*-Ethyl-cholesta-5,22(*E*)-dien-3*β*-ol (42)	Sterol	—	Seldes et al. (1988)
Cholesta-5,7-dien-3*β*-ol (60)	Sterol	—	Seldes et al. (1988)
Cholesta-5,24-dien-3*β*-ol (61)	Sterol	—	Seldes et al. (1988)
24*ξ*-Ethyl-5*α*-cholest-22(*E*)-en-3*β*-ol (62)	Sterol	​	Seldes et al. (1988)
24ξ-Ethyl-5*α*-cholestan-3*β*-ol (63)	Sterol	​	Seldes et al. (1988)
Cholest-5-en-3*β*-hydroxy-7-one (64)	Sterol	​	Seldes et al. (1988)
*T. dirhaphis*			
Tetradecanoic acid (65)	fatty acid	—	Rod′kina (2005)
Pentadecanoic acid (66)	fatty acid	—	Rod′kina (2005)
Palmitic acid (67)	fatty acid	—	Rod′kina (2005)
Margaric acid (68)	fatty acid	—	Rod′kina (2005)
Stearic acid (69)	fatty acid	—	Rod′kina (2005)
Nonadecanoic acid (70)	fatty acid	—	Rod′kina (2005)
Arachidic acid (71)	fatty acid	—	Rod′kina (2005)
Dicosanoic acid (72)	fatty acid	—	Rod′kina (2005)
Lignoceric acid (73)	fatty acid	—	Rod′kina (2005)
Octadec-6-enoic acid (74)	fatty acid	—	Rod′kina (2005)
Hexadec-9-enoic acid (75)	fatty acid	—	Rod′kina (2005)
Heptadec-9-enoic acid (76)	fatty acid	—	Rod′kina (2005)
Eicosa-9-enoic acid (77)	fatty acid	—	Rod′kina (2005)
Hexacosa-9-enoic acid (78)	fatty acid	—	Rod′kina (2005)
Hexadec-11-enoic acid (79)	fatty acid	—	Rod′kina (2005)
Octadec-11-enoic acid (80)	fatty acid	—	Rod′kina (2005)
Nonadec-11-enoic acid (81)	fatty acid	—	Rod′kina (2005)
Eicosa-11-enoic acid (82)	fatty acid	—	Rod′kina (2005)
Dicosa-15-enoic acid (83)	fatty acid	—	Rod′kina (2005)
Tetracosa-15-enoic acid (84)	fatty acid	—	Rod′kina (2005)
Hexacosa-15-enoic acid (85)	fatty acid	—	Rod′kina (2005)
Eicosa-17-enoic acid (86)	fatty acid	—	Rod′kina (2005)
Tetracosa-17-enoic acid (87)	fatty acid	—	Rod′kina (2005)
Hexacosa-17-ecoic acid (88)	fatty acid	—	Rod′kina (2005)
10,14-Methyl-pentadec-6-enoic acid (89)	fatty acid	—	Rod′kina (2005)
Octadec-8-enoic acid (90)	fatty acid	—	Rod′kina (2005)
16-Methyl-nonadecanoic acid (91)	fatty acid	—	Rod′kina (2005)
Eicosa-14-enoic acid (92)	fatty acid	—	Rod′kina (2005)
Eicosa-5,8,11,14-tetraenoic acid (93)	fatty acid	—	Rod′kina (2005)
Eicosa-5,8,11,14,17-pentaenoic acid (94)	fatty acid	—	Rod′kina (2005)
Dicosa-13-enoic acid (95)	fatty acid	—	Rod′kina (2005)
Dicosa-16-enoic acid (96)	fatty acid	—	Rod′kina (2005)
Dicosa-4,7,10,13,16,19-hexaenoic acid (97)	fatty acid	—	Rod′kina (2005)
Hexacosa-19-enoic acid (98)	fatty acid	—	Rod′kina (2005)
Hexacosa-5,9-dienoic acid (99)	fatty acid	—	Rod′kina (2005)
Heptacosa-5,9-dienoic acid (100)	fatty acid	—	Rod′kina (2005)
Hexacosa-5,9,19-trienoic acid (101)	fatty acid	—	Rod′kina (2005)
Octacosa-5,9,19-trienoic acid (102)	fatty acid	—	Rod′kina (2005)
Octacosa-5,9,21-trienoic acid (103)	fatty acid	—	Rod′kina (2005)
Octacosa-5,9,23-trienoic acid (104)	fatty acid	—	Rod′kina (2005)
14-Methy-pentadecanoic acid (105)	fatty acid	—	Rod′kina (2005)
13-Methy-pentadecanoic acid (106)	fatty acid	—	Rod′kina (2005)
15-Methy-hexadecanoic acid (107)	fatty acid	—	Rod′kina (2005)
16-Methy-heptadecanoic acid (108)	fatty acid	—	Rod′kina (2005)
15-Methy-heptadecanoic acid (109)	fatty acid	—	Rod′kina (2005)
20-Methy-heneicosanoic acid (110)	fatty acid	—	Rod′kina (2005)
19-Methy-heneicosanoic acid (111)	fatty acid	—	Rod′kina (2005)
17,18-Methy-nonadecanoic acid (112)	fatty acid	—	Rod′kina (2005)
*Tedania* sp			
2-Phenylacetamide (4)	Aromatic amide	Cytotoxic, and antibacterial	Guo et al. (2024)
Cyclo(_L_-Pro-_L_-Leu) (13)	Diketopiperazine alkaloid	—	Zhang et al. (2020)
Cyclo(_L_-Pro-_L_-Ala) (15)	Diketopiperazine alkaloid	—	Zhang et al. (2020)
Cholest-5,22-(*E*)-dien-3*β*-ol (19)	Sterol	—	De Rosa et al. (2006)
Cholest-5-en-3*β*-ol (20)	Sterol	—	De Rosa et al. (2006)
24*ξ*-Ethyl-cholest-5-en-3*β*-ol (22)	Sterol	—	De Rosa et al. (2006)
24-Nor-cholest-5,22(E)-dien-3*β*-ol (39)	Sterol	—	De Rosa et al. (2006)
Cholest-5,22-(Z)-dien-3*β*-ol (40)	Sterol	—	De Rosa et al. (2006)
24-Methyl-cholest-5,24(28)-dien-3*β*-ol (41)	Sterol	—	De Rosa et al. (2006)
24*ξ*-Ethyl-cholesta-5,22(*E*)-dien-3*β*-ol (42)	Sterol	—	De Rosa et al. (2006)
Tetradecanoic acid (66)	fatty acid	—	De Rosa et al. (2006)
Pentadecanoic acid (67)	fatty acid	—	De Rosa et al. (2006)
Palmitic acid (68)	fatty acid	—	De Rosa et al. (2006)
Margaric acid (69)	fatty acid	—	De Rosa et al. (2006)
Stearic acid (70)	fatty acid	—	De Rosa et al. (2006)
Nonadecanoic acid (71)	fatty acid	—	De Rosa et al. (2006)
Arachidic acid (72)	fatty acid	—	De Rosa et al. (2006)
Dicosanoic acid (73)	fatty acid	—	De Rosa et al. (2006)
Lignoceric acid (74)	fatty acid	—	De Rosa et al. (2006)
Bengamide B (113)	Azepine	Cytotoxic, and antibacterial	Quan et al. (2019)
Tedanizaine A (114)	Diketopiperazine alkaloid	Cytotoxic	Zhang et al. (2020)
Cyclo(_L_-Pro-_L_-Pro) (115)	Diketopiperazine alkaloid	—	Zhang et al. (2020)
Cyclo(_L_-Pro-_L_-Gly) (116)	Diketopiperazine alkaloid	—	Zhang et al. (2020)
Cyclo(_L_-Pro-_L_-Thr) (117)	Diketopiperazine alkaloid	—	Zhang et al. (2020)
Cyclo(_L_-Pro-_L_-Tyr) (118)	Diketopiperazine alkaloid	—	Zhang et al. (2020)
Tedanine (119)	Indole alkaloid	Cytotoxic, and antibacterial	Guo et al. (2024)
(3*R*)-1,3-Dihydro-3-hydroxy-3-methyl-2*H*-indol-2-one (120)	Indole alkaloid	Cytotoxic, and antibacterial	Guo et al. (2024)
Methyl-6-hydroxy-1*H*-indole-3-carboxylate (121)	Indole alkaloid	Cytotoxic, and antibacterial	Guo et al. (2024)
Loliolide (122)	Monoterpenoid	Cytotoxic, and antibacterial	Guo et al. (2024)
3-Phenylpropanamide (123)	Aromatic amide	Cytotoxic, and antibacterial	Guo et al. (2024)
Phenylacetic acid (124)	Aromatic amide	Cytotoxic, and antibacterial	Guo et al. (2024)
1-(2-Deoxy-*α*-*D*-erythro-pentofuranosyl)-1*H*-1,2,4-triazole (125)	Azole heterocyclic nucleoside	Cytotoxic, and antibacterial	Guo et al. (2024)
1-(*β*-*D*-ribofuranosyl)-1*H*-1,2,4-triazole (126)	Azole heterocyclic nucleoside	Cytotoxic, and antibacterial	Guo et al. (2024)
Dihydrouridine (127)	Pyrimidine nucleoside	Cytotoxic, and antibacterial	Guo et al. (2024)
24-Nor-cholest-5-en-3*β*-ol (128)	Sterol	​	De Rosa et al. (2006)
24*ξ*-Methyl-cholest-5,22-dien-3*β*-ol (129)	Sterol	​	De Rosa et al. (2006)
Cholest-4-en-3-one (130)	Sterol	​	De Rosa et al. (2006)
22,23-Cyclopropyl-cholest-5-en-3*β*-ol (131)	Sterol	​	De Rosa et al. (2006)
24-Ethyl-cholest-5,24(28)-(*E*)-dien-3*β*-ol (132)	Sterol	​	De Rosa et al. (2006)
24-Ethyl-cholest-5,24(28)-(*Z*)-dien-3*β*-ol (133)	Sterol	​	De Rosa et al. (2006)
23*ξ*-Methyl-cholest-5-en-3*β*-ol (134)	Sterol	​	De Rosa et al. (2006)
24*ξ*-Ethyl-cholest-5,23-dien-3*β*-ol (135)	Sterol	​	De Rosa et al. (2006)

Caption: (•), The biological activity was not evaluated.

**TABLE 3:** Biological evaluation of fractions and extracts of sponges of the genus *Tedania*.

Species	Extracts/Fractions	Tested against	References
±Cytotoxic ± Effects			
*Tedania* sp	Ethanol:water (1:1) Fractionated using water: acetonitrile (1:1) (F1 to F16)	HCT116	Banias and Uy (2023)
*T. ignis*	Ethanol, water and methanol/toluene	A549, HCT-8, PC-3 VERO and HT29	Lhullier et al. (2020), Monks et al. (2002)
*T. charcoti*	Ethanol (96%)	V-79–379 A (V-79), CaCo-2 and HeLa	Berne et al. (2015)
*T. oxeata*	Ethanol (96%)	V-79–379 A (V-79), CaCo-2 and HeLa	Berne et al. (2015)
Antimicrobial and anti-larval effects			
*Tedania* sp	methanol and ethanol (95%) F10 a 12	*M. tuberculosis* H37Rv (ATCC27294*), E. faecalis* and *A. aegypti*	Quan et al. (2019)
*T. ignis*	Ethanol, water and methanol/toluene	*E. faecalis* (ATCC 29212). *P. aeruginosa* (ATCC 27853), *E. coli* (ATCC 25922), *L. amazonensis*, *T. cruzi,* HSV-1 (cepa KOS). *S. aureus* (ATCC 6538P)*, S. epidermidis* (ATCC 12228)*, B. subtilis* (ATCC 6633), *M. luteus* (ATCC 9341), *C. albicans* (ATCC 10231) and *S. cerevisiae* (ATCC 1600)	Lhullier et al. (2020), Monks et al. (2002)
*T. stylonychaeta*	Ethyl acetate and dichloromethane:methanol	Methicillin-resistant *S. aureus* ATCC 49476, *P. aeruginosa* ATCC 10145, *C. difficile* ATCC 9689, *C. albicans* ATCC 10231 and *A. fumigatus* ATCC 204305	Kibungu et al. (2021)
*T. massa*	Ethanol (96%)	*Pseudomonas* spp.(ARK 13, ARK 14, ARK 285); *P. aeruginosa* multi-resistant (12,599); *E. coli* (DH5; 3273; Z39; KM 128), TL 747, *S. enterica ser* (TL 747), *Typhimurium; K. pneumonia* (KPC 1705), *A. baumannii* (12,588) *Rhodosporidium lusitanie* (EX-3935) *Debaryomyces hansenii* (EX-4023) *Rhodotorula mucilaginosa* (EX-4015) *Cryptococcus carnescens* (EX-1551), *Aureobasidum subglaciale* (EX-2481) *Candida albicans* (EX-9382), *Candida parapsilosis* (EX-9370), *Exophiala dermatitidis* (EX-5721), *Fusarium dimerum* (EX-9424), *Aureobasidum melanogenum* (EX-9454) and *A. melanogenum* (EX-9467)	Berne et al. (2015)
*T. charcoti*	Ethanol (96%)	*Pseudomonas* spp. (ARK 13, ARK 14, ARK 285), *P. aeruginosa* multi-resistant (12,599), *E. coli* (DH5; 3273; Z39; KM 128), TL 747, *S. enterica ser* (TL 747), *Typhimurium*, *K. pneumonia* (KPC 1705), and *A. baumannii* (12,588)	Berne et al. (2015)
*T. oxeata*	Ethanol (96%)	*Pseudomonas* spp. (ARK 13, ARK 14, ARK 285), *P. aeruginosa* multi-resistant (12,599), *E. coli* (DH5; 3273; Z39; KM 128), TL 747, *S. enterica ser* (TL 747), *Typhimurium; K. pneumonia* (KPC 1705), and *A. baumannii* (12,588)	Berne et al. (2015)
Anti-inflammatory effect			
*T. ignis*	Ethanol (50%):water and acetone	MAPK/ERK incubated in SW-13 cells	Brown et al. (2001)
		Proteolysis, and hemolysis	Faioli et al. (2013)

#### 3.1.1 *Tedania ignis* duchassaing and michelotti, 1864

**FIGURE 3:**
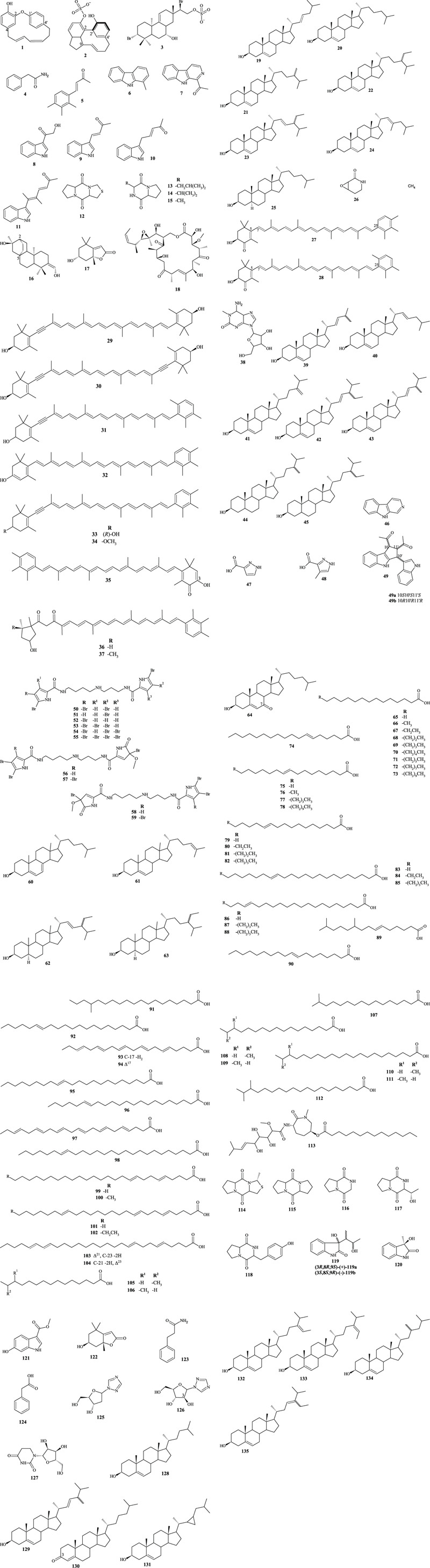
Chemical compounds isolated from marine sponges of the genus *T*.*edania*.

Among the species of the genus *Tedania*, *Tedania ignis* is the most chemically investigated. The most recent report on this species describes the isolation and purification of diarilheptanoids **1** and **2**, obtained by chromatographic fractionation eluted with methanol:water (8:2) from combined butanol:chloroform extracts. Structurally, diarilheptanoid **1** exhibited aromatic rings interconnected at C-2 and C-1′ by an ether linkage and connected at C-4 and C-4′ by a homogeneous diunsaturated seven-carbon chain, forming a third ring. Metabolite **2** presented a phenolic group fused to a cyclopentane ring and a benzenesulfonic acid moiety. The benzene units are linked via C-2 and C-2′ through an endocyclic sp^3^ bond, and the cyclopentane unit is linked to the benzenic acid group via C-9 and C-4′, bridged by a homogeneous unsaturated four-carbon chain, forming a tetracyclic skeleton (Costantino et al., 2012). Additionally, a tricyclic ent-pimarane diterpenoid **3**, was also isolated under the same chromatographic conditions as the diarilheptanoids **1** and **2** (Costantino et al., 2009), as shown in Figure 3.

In the study by Dillman and Cardellina (1991b), the isolation of several compounds from the fraction hexane:dichloromethane:ethyl acetate (4:3:1) was reported: benzene derivatives **4** (monosubstituted) and **5** (tetrasubstituted), carbazole **6**, *β*-carboline derivative **7**, and indolic alkaloids **8** to **11**, which share identical heterocyclic cores but differ in the acyclic side chains. Despite their identification, the authors suggest that indoles **9** and **10** are artifacts generated by the condensation of acetone solvent with indolic aldehydes.

Another class of metabolites identified were piperazines, especially diketopiperazines **12** to **15**, as well as diterpenes **16** (atisane diterpenoid) and **17** (terpene lactone). Diketopiperazines are a class of heterocyclic metabolites resulting from the condensation of two amino acids, exhibiting a central lactam common to all members of this class. metabolite **12** showed two five-membered rings connected on either side of the central lactam, forming a 5/6/5 junction. Metabolite **13** to **15** shared the same cyclic structure but differed in the length of the branched chain (Dillman and Cardellina, 1991a; Schmidtz et al., 1983). The authors also described diterpenoid **16**, a heterocycle with a 4/6/6/6 ring fusion, where the four-membered ring is connected to the adjacent ring via C-2 and C-5 and contains a hydroxyl group. Additionally, a heterocyclic monoterpenoid **17** with a 6/5 ring fusion containing a lactone and a hydroxyl group was reported (Schmidtz et al., 1983).

Another important metabolite reported in *T. ignis* was macrolide **18**, obtained from the chloroform:methanol (1:1) extract, which featured a 32-carbon chain with two sp^2^ bonds. This marine macrolide exhibited a highly diverse heterocyclic identity, with lactone, epoxide, methoxy, hydroxyl, and ketone groups, distinguishing itself from others due to lactonization occurring between oxygenated groups rather than carbon branches (Schmitz et al., 1984).

In the more apolar fractions (hexane:ethyl acetate) of *T. ignis*, seven sterols **19** to **25** were identified. These sterols exhibited the same cyclic skeleton with a 6/6/6/5 ring fusion, differing in the branching of the acyclic side chain and types of bonds. Furthermore, sterols **19** to **24** exhibited an sp^2^ bond at C-5, whereas sterol **25** lacked any unsaturation. Quantitative analysis revealed sterol **25** as the major metabolite (33.19%) of this fraction (Montaño et al., 2009). Regarding the more polar fractions (hexane: dichloromethane:ethyl acetate), *δ*-lactam **26** was elucidated, previously known only synthetically and reported for the first time from a natural source. The chemical structure of **26** displayed cyclic amide and epoxide groups forming a heterocyclic two-ring chain (Cronan and Cardellina, 1994).

#### 3.1.2 *Tedania digitata* Schmidt, 1862

Between 1977 and 2001, *Tedania digitata* was the focus of research by Tanaka and collaborators, who reported several compounds belonging to the carotenoid class. This species has also been studied by other authors; however, no recent findings have been reported.

From the acetone extract of *T. digitata*, xanthophylls carotenoids **27** and **28** were isolated and identified. These carotenoids exhibited similar structures, consisting of two six-membered rings connected at C-6 and C-25 by an 18-carbon acyclic chain, differing in the orientation of the substituent on the aromatic ring. Carotenoids **27** and **28** were identified as xanthophylls, resulting from oxidation processes that generated hydroxyl and ketone groups on one of the rings (Tanaka et al., 2001; Uenojo and Maróstica, 2007).

Additional xanthophylls carotenoids, **29** to **35**, were identified from *T. digitata* extracts. These metabolites showed structural similarities, differing in the degree of unsaturation at C-7 and C-23 and/or the nature of the oxygenated functional groups present (Tanaka and Katayama, 1979; Tanaka et al., 1977b; Saxegaard et al., 1981; Tanaka and Katayama, 1980; Tanaka and Inoue, 1988; Okukado, 1975; Tanaka et al., 1978). Furthermore, metabolites **29** and **30** lacked an aromatic ring (Saxegaard et al., 1981). Carotenoid **35** exhibited a structure similar to **27**, differing by the presence of an sp^2^ unsaturation at C-3 (Okukado, 1975; Tanaka et al., 1978). In this investigation, carotenoid **35** was identified as the major metabolite of the petroleum ether fraction, comprising 37% of the total composition (Tanaka et al., 1978).

In an earlier study by Tanaka et al. (1977a) two ketocarotenoids, **36** and **37**, were isolated from a acetone extract. These compounds had similar structures, differing by the presence of a methyl group in the cyclopentane unit. Additionally, nucleoside **38** was identified from the ammonium-eluted fraction. This nucleoside was characterized as a methylated purine containing a ketone group between tertiary amines (Quiann et al., 1980).

#### 3.1.3 *Tedania anhelans* Keller, 1891

Chemical studies using ethyl acetate and methanol extracts of *Tedania anhelans* enabled the identification of various compounds, including lipids, alkaloids, and nitrogenous acids. Among these classes, sterols **20**, **21**, **22**, **25**, and **38** to **45** were identified, exhibiting structural similarities characterized by four fused rings with a 6/6/6/5 junction, although differing in the spatial orientation of the acyclic side chain, types of bonds, and the presence or absence of branching. Moreover, sterols **44** to **45** lacked sp^2^ unsaturation at C-3. Regarding the major metabolites of the ethyl acetate extracts from *T. anhelans* collected from both lagoon and open sea environments, the relative abundances of the following sterols were quantified: **20** (31.8% and 35.1%), **25** (20.1% and 19.4%), **41** (11.4% and 10.0%), and **22** (9.5% and 10.2%), respectively (Aiello et al., 1993).

In studies with the methanol extract of *T. anhelans*, aromatic heterocyclic metabolites **46** to **49** were isolated. Carbazole **46** featured two aromatic rings and a cyclopentane with nitrogenous functional groups. In contrast, carboxylic acid **47** and **48** contained only one pyrazole ring, bearing acidic functional group (Parameswaran et al., 1997). Another substance identified in the methanol extract of this species was the novel racemic alkaloid **49**, isolated from the methanol:water (7:3) subfraction, which exhibits two indole groups and two ketone units in its chemical structure (Jin et al., 2021).

#### 3.1.4 *Tedania brasiliensis* mothes, Hadju and Van soest, 2000

In a study conducted by Parra et al. (2018) bromopyrrole alkaloid **50** was isolated and identified from the hexane, ethyl acetate, and water fractions, and bromopyrrole alkaloid **51** to **59** were found in the ethyl acetate fractions of *T. brasiliensis*. These compounds are alkaloids with mixed heterogeneous chains, featuring a heterocyclic group at each end of the chain. Alkaloids **51**–**52**, **53**–**54**, **56**–**57**, and **58**–**59** are inseparable isomeric mixtures, differentiated by their bromination sites.

#### 3.1.5 *Tedania excavata* Thiele, 1905

In the more apolar extracts of *T. excavata*, thirteen sterols (**19** to **22**, **39**, **41 to 43**, and **60**–**64**) were identified. These compounds exhibited structures typical of phytosterols, with fused rings arranged in a 6/6/6/5 junction, differing in the branching of the acyclic side chain, spatial orientation, bond types, and degree of unsaturation in the cyclic chain. Cholesterol **64** presented a ketone group at C-7, distinguishing it from the others. Sterols **20**, **22**, and **39** were obtained from the petroleum ether:ethyl acetate fraction (increasing polarity) and were the major metabolites, representing 49.5%, 16.1%, and 11.3%, respectively (Seldes et al., 1988).

#### 3.1.6 *Tedania dirhaphis* Hentschel, 1912

Reported a collection of **47** fatty acids, **65** to **112**, from the chloroform:methanol (2:1) extract of *T. dirhaphis*. The majority of these compounds (29 of them) had carbon chains ranging from 14 to 28 carbons, with 1–6 degrees of unsaturation. The remaining 19 metabolites were saturated fatty acids with chain lengths between 14 and 24 carbons. Fatty acid **103** (63.3%) was the major metabolite of this extract. Fatty acids **74**, **89** to **91**, and **93** were reported for the first time in marine sponges (Rod′kina, 2005).

#### 3.1.7 *Tedania* sp.

There are reports in the literature where species of the genus *Tedania* were not identified; therefore, the compounds are attributed to the specimen. In the study by Quan et al. (2019), the methanol extract of *Tedania* sp. was purified using an acetonitrile:water gradient (0%–100%), producing 12 fractions. Fraction 12 was obtained in its purest form, which enabled the elucidation of its chemical structure, identified as azepine **113**. This metabolite exhibited multiple oxygenated and nitrogenated functional groups such as methoxy, hydroxyls, ester, and amides, featuring a mixed 32-carbon chain.

In a study with the ethanol 9% extract of *Tedania* sp., followed by a petroleum ether:ethyl acetate gradient, Zhang et al. (2020), isolated seven thiodiketopiperazines, metabolites **13**, **15** and **114** to **118**, including one previously unknown. This class of metabolites is characterized by a heterocyclic chain of two or three rings containing a diketopiperazine core. This core is common to all metabolites, and they differ in secondary groups, such as sulfur fused in one of the cycles, monophenol, hydroxylated branching, or hydrocarbon chains. Compounds **13** and **15** have been previously reported in *T. ignis*, and the other metabolites showed structural similarity to those described in this species (Dillman and Cardellina, 1991b).

Fractionation of the ethyl acetate extract of *Tedania* sp. using a chloroform:methanol gradient yielded seven fractions, from which the indole alkaloid **119** was isolated for the first time, along with compounds **4**, **120** to **127**. Alkaloid **119** exhibited an aromatic ring linked to a lactam and another acyclic portion. Alkaloid **120** showed structural similarity to **119** but differed by having a methyl substituent instead of a mixed chain. Alkaloid **121** presented a phenol group as well as an ester function. The monoterpenoid **122**, an isomer of metabolite **17** previously isolated from *T. ignis*, exhibited an unsaturated lactone and a fused cyclohexanol. Aromatic metabolites **4**, **123** and **124** displayed similar structures and compositions, varying between 8 and 9 carbons atoms and containing either amide or acid functional groups. Nucleoside **125** and **126** are similar, differing by the presence of a hydroxyl group at C-3 in the polyhydroxylated cyclic ether. Meanwhile, nucleoside **127** featured two rings connected by an sp^3^ carbon-nitrogen bond, and also contained lactam groups (Guo et al., 2024).

A total of 24 metabolites were isolated and identified from chloroform extracts of two specimens of *Tedania* sp. The purification of these metabolites using petroleum ether:acetone gradients and chloroform:methanol gradients, as well as petroleum ether:acetone (10:1) and chloroform, yielded sterols **19**, **20**, **39** to **43**, and **128** to **135**, and fatty acids **65** to **73**. These sterols exhibited similar structures and functional groups, differing in degree of unsaturation and stereochemistry (De Rosa et al., 2006). Metabolites **19** and **20** were later described in *T. ignis*, sterols **39**, **41** to **43** had been previously reported in *T. anhelans* (Aiello et al., 1993) and *T. excavata* (Seldes et al., 1988); and fatty acids **65** to **73** in *T. dirhaphis* sponges (Rod′kina, 2005). Sterol **130** showed a structure similar to the others, although it results from oxidation of the secondary alcohol at C-3. Cholesterol **131** presented the same heterocyclic skeleton as the others but differed by the presence of a ring in the acyclic side chain (De Rosa et al., 2006).

### 3.2 Biological activities of species of the genus *Tedania*

Regarding the biological activities of species of the genus *Tedania*, reports indicate cytotoxic, anti-inflammatory, antimicrobial, and larvicidal activities. Anti-inflammatory activity involves signaling pathways associated with induction proliferation and inhibition apoptosis in cancer cells (Costantino et al., 2009; Jin et al., 2021), as well as enzymes responsible for prostaglandin production, which play a role in pain reduction (Costantino et al., 2009), among other effects.

Concerning cytotoxic activity, studies have predominantly focused on cancer cell lines affecting both males and females, including prostate cancer (*PC-3*), cervical cancer (*HeLa*), leukemia (*K562*; *RAW 264.7*), pancreatic cancer (*ASPC-1*), lung cancer (*A549*), colorectal carcinoma (*HCT116*; *HCT-8*; *HT29*; *CaCo-2*), liver cancer (*HepG2*), and renal cancer (*HEK293*). The metabolites were investigated for their antiproliferative capacity, toxicity against normal cells (*VERO*; *V79*) (Lhullier et al., 2020; Berne et al., 2015) and mechanisms underlying antiproliferative activity using the Caspase-Glo® 3/7 assay (Banias and Uy, 2023), in *in vitro* assays, as well as for their effects on both pro- and anti-angiogenic pathways (Jin et al., 2021; Guo et al., 2024), in *in vivo* assay.

With respect to antimicrobial and larvicidal activities, investigations targeted microorganisms responsible for tuberculosis (Quan et al., 2019), leishmaniasis (Lhullier et al., 2020), Chagas disease (Parra et al., 2018), various infections (Guo et al., 2024), herpes (Lhullier et al., 2020) and dengue (Wantah et al., 2018).

Below is a compilation of biological activities related to chemical substances, fractions, and extracts obtained from marine sponge species of the genus *Tedania*.

#### 3.2.1 Cytotoxic effects

Uncontrolled cell growth can occur in any part of the body and is generally associated with various external factors (such as lifestyle habits, infections, exposure to carcinogenic metabolites, etc.) and internal factors (including DNA alterations, cell cycle disruption, gene inactivation, etc.). The implication of these factors initially causes effects localized at the site of origin (primary tumor), but if not diagnosed and treated early, the condition may spread throughout the body and cause metastasis (the development of secondary tumors) (Brandão et al., 2010). In this context, the present study includes the investigation of 13 cancer cell lines and 2 normal cell lines, derived from human and animal sources, which were evaluated against pure metabolites, fractions, and extracts from species of the genus *Tedania*.

Throughout the study, alkaloids were identified as the primary class under cytotoxic analysis. Jin et al. (2021), investigated the racemic alkaloids **49a** and **49b** for their antiproliferative potential against *HCT116*, *HeLa*, *ASPC-1*, and *K562* cell lines using the 3-(4,5-dimethylthiazol-2-yl)-2,5-diphenyltetrazolium bromide (MTT) and sulforhodamine B (SRB) assays. Additionally, their pro- and anti-angiogenic effects were evaluated *in vivo* using zebrafish model with Green Fluorescent Protein (GFP) and Enhanced Green Fluorescent Protein (EGFP) fluorescence markers, as well as the Intersegmental Vessel (ISV) assays, with adriamycin used as the positive control.

These metabolites showed limited activity against all tested cell lines, with IC_50_ values of 2.2 μM (*K562*) for alkaloid **49a**, and 7.1 μM (*HCT116*) and 9.9 μM (*HeLa*) for alkaloid **49b**. In contrast, adriamycin exhibited IC_50_ values of 0.21 μM (*HCT116*), 0.60 μM (*HeLa*), 0.86 μM (*ASPC-1*), and 0.25 μM (*K562*). However, both alkaloids demonstrated stronger pro-antiangiogenic effect, with length of around 2000 ISV/μm compared to the positive control (Danhong injection), which had length above 3000 ISV/μm, at the same concentrations (20 μg mL^-1^) (Jin et al., 2021).

Another group of alkaloids, **50** and a mixture of metabolite **51**–**52**, was studied for their cytotoxic potential against *HepG2* cells using the MTT assay at varying sample concentrations (1–1000 μg mL-1). Both metabolites were inactive against this cell line, with LD_50_ values of 16 ± 1 μM and ≥400 μM, respectively. No morphological alterations were observed in *HepG2* cells treated with 10 μM of compound **50** after 24 h (Parra et al., 2018).

In a separate investigation, fractions 10 to 12 and metabolite **113** (3.9 μg mL^-1^–500 μg mL^-1^) from *Tedania* sp. were assessed for their ability to reduce the viability of *HepG2* (ATCC HB-8065), *THP-1* (ATCC TIB-202), *HEK293* (ATCC CRL-1573), and *A549* (ATCC CCL-185) cell lines, cell viability was determined by the resazurin reduction assay. Although compound **113** inhibited the MetAP1 enzyme in bacteria, no inhibition of proliferation was observed in the tested tumor cell (Quan et al., 2019). The thiodiketopiperazine **114** was also evaluated against *A549* cells and murine macrophages (*RAW 264.7*), but no cytotoxic effects were detected (Zhang et al., 2020).

Various isolated metabolites, including alkaloids **119**, **120**, **121**, monoterpenoid **122**, aromatic amides **4** and **123**, and aromatic acid **124**, and nucleoside **125** to **127** were also evaluated for their ability to inhibit proliferation of *K562* cells. Although alkaloids are generally known for anticancer activity, the metabolites mentioned in this study, along with others isolated from *Tedania* sp., did not demonstrate this effect (Guo et al., 2024).

In the study by Banias and Uy (2023), the authors evaluated the antiproliferative and pro-apoptotic activities of fractions obtained from ethanol:water extract of *Tedania* sp. Fractionation was performed by gradient elution using water, acetonitrile, and ethyl acetate. A total of 16 fractions were obtained and tested against *HCT116* cell lines using the MTT assay in an *in vitro* experiment. A volume of 10 µL of the sample (300  μg mL^-1^) was added to wells containing 20,000 HCT116 cells in (95 µL), and the results were compared with positive (digitonin) and negative (0.3% DMSO) controls. Fraction 16 (ethyl acetate 100%) reduced *HCT116* cell viability to 90.9%, compared with the positive control, which reduced cell viability to 25%.

Regarding pro-apoptotic assays, the fractions (300 μg mL^-1^) were tested using the Caspase-Glo® 3/7 assay in 10,000 *HCT116* cells cultured in 96-well plates. Sodium butyrate was used as the positive control, while DMSO (0.3%) served as the negative control. The fractions obtained using thr water:acetonitrile 8 (6:4), 9 (2:8), and fraction 10 (100% acetonitrile) exhibited significant fold change (FC) values compared with sodium butyrate, which showed an FC of 4.3. Among them, fraction 9 stood out, with an FC of 2.98, suggesting induction of pro-apoptotic activity through caspase 3/7 (Banias and Uy, 2023). Therefore, the antiproliferative effect observed in fraction 16 did not occur through caspase 3/7 activation.

An ethanolic extract of *T. ignis* was tested against *A549*, *HCT-8*, *PC-3*, and normal *VERO* cell line using the SRB method. The extract reduced the cell viability of the four tested cell lines, with IC_50_ values (µg mL^-1^) of 30.2, 6.5, 17.8, and 37.9, respectively. For comparison, paclitaxel showed IC_50_ values of 0.14, 0.18, 0.57, and >500, respectively. Despite the potent antiproliferative effect of the extract, no selectivity toward cancer cells was observed (Lhullier et al., 2020).

Extracts of *T. ignis* methanol:toluene (3:1) and water, at a concentration of 100 μg mL^-1^, were tested against *HT29* cells, using the SRB assay, showing cell viability of 44% and 90%, respectively, compared to the control group (0.25% DMSO). Although both extracts were tested against *HT29* cells, the organic extract demonstrated higher cytotoxic potential than the aqueous extract (Monks et al., 2002).

In the study by Berne et al. (2015), evaluated toxicity and cell viability induced by 96% ethanolic extracts (100 µg seco mL^-1^) of *T. charcoti* (EETc) and *T. oxeata* (EETo) in *CaCo-2* (10,000 cells per well), *HeLa* (5,000 cells per well), and normal *V79* (5,000 cells per well) cells using the 3-[4,5-dimethylthiazol-2-yl]-5-[3-carboxymethoxyphenyl]-2-[4-sulfophenyl]-2H-tetrazolium (MTS) assay, with ethanol as the control. EETc and EETo showed 97% and 54.4% viability in *CaCo-2* cells, respectively, with no effects detected in *HeLa* cells. Both extracts were selective for *CaCo-2* adenocarcinoma cells, not reducing viability in normal *V79* cells (105.1% and 94.6% viability, respectively).

#### 3.2.2 Antimicrobial

Pathogenic microorganisms attract scientific interest due to the numerous infections they cause and their high contamination potential. This study encompasses antimicrobial effects against standard and multidrug-resistant bacteria, including Gram-positive, Gram-negative, and *Mycobacterium tuberculosis* (Koch’s bacillu*s*), fungi from the classes Saccharomycetes and Eurotiomycetes, parasites responsible for leishmaniasis and Chagas disease, herpesviruses (Lhullier et al., 2020; Quan et al., 2019; Parra et al., 2018).

Alkaloids **50**, **51**–**52**, and **56**–**57** were investigated through *in vitro* parasite growth inhibition assays against *Plasmodium falciparum* (3D7 sensitive and K1, chloroquine-, cycloguanil, and pyrimethamine-resistant strains), *Leishmania* (L.) *amazonensis*, *Leishmania* (L.) *infantum*, and *Trypanosoma cruzi*. All alkaloids were inactive, with CE_50_ > 100 µM for all parasites tested, except compounds **50** and **51**–**52**, which exhibited CE_50_ values of 1.1 ± 0.1µM and 5.8 ± 0.5µM, respectively, against *P. falciparum* (3D7). Alkaloid **50** demonstrated antiplasmodial activity against drug-resistant strains of *P. falciparum* (K1), with an IC_50_ of 1.1 ± 0.1 µM (Parra et al., 2018). This effect may be related to the presence of an additional bromine atom in alkaloid **50**.

In the study by Quan et al. (2019), the antimycobacterial potential of 12 acetonitrile:water fractions (gradient from 0% to 100%) and of metabolite **113** was evaluated, both from *Tedania* sp., against *Mycobacterium tuberculosis* strains resistant to isoniazid, rifampicin, and ethambutol. Bacterial viability assays identify fractions 10 to 12 (50 μg mL^-1^) as the most potent, with MIC_50_ values of 0.156, 0.078, and 0.078 μg mL^-1^, respectively, relative to untreated control wells. These fractions were also tested in intracellular assays using *THP-1* macrophages infected with *M. tuberculosis* (*H37Rv*), confirming their inhibitory potential, with a reduction from 2.5 × 10^4^ to 5 × 10^4^ *THP-1* cells per well containing 10^6^ *H37Rv* bacteria after 4 h of treatment at 20 μg mL^-1^

Subsequently, the capacity of metabolite **113** (5 µM) to inhibit methionine aminopeptidase (*MetAP*) activity from *M. tuberculosis* (*MetAP1c*) and *Enterococcus faecalis* (*MetAP1b*) enzymes was evaluated, através de ensaios *in vitro*, showing potent inhibitory effects with inhibition potentials of 72.84%, and 83.33%, respectively. Although a strong inhibitory effect on *MetAP* was observed, this effect did not translate into inhibition of *E. faecalis* growth *in vitro*. Furthermore, azepine **113**, tested at an initial concentration of 1 µM in combination with rifampicin (1 nM) and subjected to 1:3 serial dilutions, demonstrated strong synergy against *M. tuberculosis,* enabling more than a 200-fold reduction in rifampicin dosage and over a 14-fold reduction for azepine **113** itself (Quan et al., 2019).

Although Quan et al. (2019) do not directly attribute antibacterial effects to metabolite **113**, it is known that *M. tuberculosis* is resistant to hydrophilic metabolites due to the impermeability of its cell wall and the presence of mycolic acids (Tanaka et al., 1978). As mentioned in item 3.1.7, metabolite **113** contains oxygenated and nitrogenated groups-such as esters, ethers, alcohols, amides, and a hydrocarbon chain consisting of 32 carbons imparting low polarity, which may favor its passage through the bacterial cell wall and inhibition of the *MetAP1* enzyme.

In the study by Guo et al. (2024), evaluated the antibacterial effects of metabolites **119** to **127** against standard strains of *E. faecalis* (ATCC29212), *Staphylococcus aureus* (ATCC 25923), *Pseudomonas aeruginosa* (ATCC27853)*, Acinetobacter baumannii* (ATCC19606), and *Escherichia coli* (ATCC25922). Despite structural and functional differences among these metabolites and bacterial cell wall variations, none exhibited significant antibacterial activity against the tested strains, except alkaloid **119**, which showed moderate activity against *E. coli* with an MIC of 64 μg mL^-1^.

In the study of Lhullier et al. (2020) the antiparasitic effect of ethanolic extract (50 μg mL^-1^) from *T. ignis* was investigated against *L. amazonensis* and *T. cruzi*. The extract was inactive, inhibiting parasite growth by 4.17% and 16.86%, respectively. In comparison, the positive controls amphotericin B (1.84 μg mL^-1^) and benznidazole (5.2 μg mL^-1^) inhibited 96.59% of *L. amazonensis* and 90.93% of *T. cruzi,* respectivety, while DMSO (1%) was used as the negative control. In addition to the antiparasitic effects, the authors evaluated anti-herpetic activity against *HSV-1* (KOS strain) using the SRB method, with acyclovir as the positive control and HSV-1 (29-R strain) as the negative control. Antibacterial activity against *E. faecalis, P. aeruginosa*, and *E. coli*, using the disk diffusion method. Ampicillin (10 µg), oxacillin (1 µg), ceftazidime (30 µg), and fluconazole (25 µg) were used as positive controls, and DMSO served as the negative control. The ethanolic extract inhibited viral replication by 40.88% after 48 h of treatment at 50 μg mL^-1^ compared with acyclovir (IC_50_ = 1.23 μg mL^-1^), but was inactive against all bacterial strains tested at 100 mg mL^-1^. All experiments were performed in 96-well microplates under *in vitro* conditions.

In the search for Monks et al. (2002), were assessed the antimicrobial potential of methanol:toluene and aqueou extracts against *E. coli*, *S. aureus*, *Staphylococcus epidermidis, Bacillus subtilis*, *Micrococcus luteus*, *Candida albicans*, and *Saccharomyces cerevisiae* using the disk diffusion method at a concentration of 2.5 mg mL^-1^, in comparison with the antibiotic chloramphenicol (400 μg mL^-1^). Both extracts were inactive against all tested microorganisms.

Berne et al. (2015) evaluated antibacterial activity of ethanolic extracts from three *Tedania* species. Extracts EETm, EETc, and EETo were tested against standard and multidrug-resistant microorganisms using the agar diffusion method. A volume of 10 µL of each extract was applied onto plates previously inoculated with 100 µL of bacterial suspensioin. The antibiotics rifampicin (25 µg), ciprofloxacin (5 µg), gentamicin (10 µg), chloramphenicol (30 µg), tetracycline (5 µg), and ampicillin (10 µg) were used as positive controls, while ethanol served as the negative control. Plates were incubated for 24 h at 18 °C or 37 °C, depending on the origin of bacterial isolation.

The extracts showed antibacterial effects against at least one strain of *Pseudomonas* spp., with inhibition zones of 9–10 mm. EETm and EETc inhibited growth of at least 1 *E. coli* strain with zones between 7 and 9 mm. Only EETc and EETo showed activity against *Salmonella enterica*, with inhibition zones of 9-8 mm, respectively. EETm and EETo exhibited inhibitory potential against *A. baumannii*, with zones of 10-9 mm, respectively. Overall, EETm and EETc inhibited a greater number of bacterial strains, mainly targeting Gram-negative bacteria (Berne et al., 2015).

The antifungal activity of the ethanolic extract EETm was also evaluated against different fungal strains using the broth dilution method for yeasts and a specific assay for filamentous fungi. An inoculum of 10^5^ cell conidia^−1^ mL^-1^ (95 µL) was used, to which 5 µL of the extract was added, resulting in a final concentration of 100 µg of dry extract mL^-1^. The assays were performed in 96-well microplates and incubated for 72 h at 25 °C. Turbidity was used as a parameter to determine antifungal activity, and results were expressed as percentage inhibition compared to the negative control. EETm showed 51%–75% inhibition against the genera *Rhodosporidium*, *Rhodotorula*, *Aureobasidium*, and *Candida*, and up to 50% inhibition against the remaining fungal strains tested (Berne et al., 2015).

On the other hand, the ethyl acetate extract of *T. stylonychaeta* was tested against MRSA *S. aureus, P. aeruginosa*, *Clostridioides difficile*, *Aspergillus fumigatus*, and *C. albicans* using the agar diffusion method. The antibiotics used as positive controls were vancomycin (30 µg), clindamycim (10 µg), trimethoprim (5 µg), rifampicin (5 µg), and amoxicillin (25 µg) for MRSA; imipenem (10 µg), ciprofloxacin (5 µg), and meropenem (10 µg) for *P. aeruginosa*; and metronidazole (5 µg), tetracycline (30 µg), and clarithromycin (15 µg) for *C. difficile*. Plates were incubated for 48 h for the anaerobic bacterium and 24 h the remaining bacteria at 37 °C. For the positive control of antifungal activity, fluconazole (15 µg), itraconazole (10 µg), and voriconazole (5 µg) were used, while the extract solvent served as the negative control. Incubation was carried out for 72–120 h at 37 °C. the extract was tested at concentrations ranging from 7.2 to 24  mg mL^-1^ (Kibungu et al., 2021).

The extract showed a MIC of 7.2  mg mL^-1^ and MBC 9.6  mg mL^-1^ for all tested microorganisms, except *P.aeruginosa*, which had a MIC of 19mg mL-1, MBC of 14.4mg mL-1, and inhibition zones of 20, 17, and 15 mm (24 mg mL^-1^), respectively. For *C. albicans*, the inhibition zone was 20 mm (24 mg mL^-1^). Positive controls showed inhibitions zones ranging from 10 to 40 mm (Kibungu et al., 2021). Although the extract showed relevant inhibition zones, the high concentrations required may represent a limitation due to potential toxicity.

#### 3.2.3 Anti-larval effects

In a study conducted by Wantah et al. (2018), the larvicidal potential of 95% ethanolic extract from *Tedania* sp. against *Aedes aegypti* larvae was evaluated. For the assay, larvae were reared from the early stages and fed until reaching the 3° instar, at which point the *in vivo* test was performed. 10 larvae were placed in each tube (triplicate), containing 10 mL of the extract at 1000ppm. Abate (1000ppm) was used as the positive control, and clean water served as the negative control. The test lasted 24 h, with observations recorded every hour. The extract exhibited significant larvicidal activity, reaching 69% larval mortality after 2 h of exposure and 100% mortality after 8 h.

#### 3.2.4 Anti-inflammatory effect

In the study by Costantino et al. (2012), phenolic metabolites were isolated, which are known for their anti-inflammatory activity and inhibition of nitric oxide (NO) production. The anti-inflammatory potential of diarylheptanoids **1** and **2** was evaluated through the measurement of nitrite (NO_2_^−^) production induced by *E. coli* LPS in murine monocyte/macrophage cell lines (*J774*). *J774* cells (density of 2.5x10^6^ cells mL^-1^) were seeded in 24-well plates and allowed to adhere for 2 h. Subsequently, the compounds at concentrations of 3. 10, and 30µM, followed by incubation for 24 h. Cell viability was determined using the MTT assay in an *in vitro* model, and NO^2-^ levels were quantified using the Griess reaction. Results were expressed relative to the positive control (*J774* cells stimulated with LPS and left untreated).

metabolite **1** demonstrated a favorable effect, inhibiting NO_2_^−^ production at concentrations of 10 and 30 µM in a dose-dependent manner. Conversely, diarylheptanoid **2** was inactive at these concentrations, and an increase in NO_2_^−^production was observed. This negative effect of compound **2** may be related to the negative charge present on the oxygen atom, which could impair or reduce its ability to stabilize the NO_2_^−^ ion (Costantino et al., 2012).

Diterpenol **3** (1 mg kg^-1^) was assessed for its anti-inflammatory potential via carrageenan-induced paw edema and myeloperoxidase (MPO) activity in mice, at different doses (0.1, 0.3, and 1 mg kg^-1^) through an *in vivo* assay. The metabolite **3**significantly reduced paw edema from 0.1 mL (vehicle) to 0.05 mL (metabolite **3**, 1 mg kg^-1^), with p < 0.001 vs. vehicle, at 4 and 48 h. It also inhibited MPO infiltration in cells at 4 and 48 h (p < 0.01 vs. vehicle) after carrageenan administration. Mechanistic evaluation, performed through *in vitro* assays, revealed that diterpenoid **3** inhibited the *COX-2* pathway at 2 and 4 h, *COX-1* at 48 and 72 h, and *iNOS* at 2 and 48 h after edema induction, showing greater affectiveness in the COX-2 pathwas. These results suggest that this compound may reduce vascular permeability and cellular migration involving these pathways (Costantino et al., 2009).

Other important signaling pathways include *HIF-1*, *Wnt*, and *STAT3/NF-κB*, which regulate gene expression related to cancer cell survival, apoptosis inhibition, proliferation, and angiogenesis, thereby increasing tumor aggressiveness (Katoh and Katoh, 2022; Nagy, 2011; Hendrayani et al., 2016), in contrast, the *PPARγ* and *p53* signaling pathways are associated with cellular stress leading to cell cycle arrest or apoptosis of cancer cells (Conte and Salles, 2002).

In the study by Jin et al. (2021), the ability of alkaloids **49a** and **49b** to inhibit the *HIF-1*, *Wnt*, and *STAT3/NF-κB,* and activation of *PPARy* and *p53* pathways were investigated using zebrafish model through an *in vivo* assay, employing the following positive controls: KC7F2 (*HIF-1*), LF3 (*Wnt*), and brevilin A (*STAT3/NF-κB*). Metabolite **49a** showed inhibition percentages higher than or close to those of the positive controls, which were 96% (20 μM), 70,9% (20 μM), and 100% (7.5 μM), respectively. Meanwhile, the metabolite **49a** exhibited inhibitory effects of 99.6%, 96,6%, and 77.1%, respectively, at 20 μM, and at 5 μM they inhibited 70.6% (*HIF-1*) and 72.4% (*Wnt*). Thus, metabolite **49a** may act by modulating the *HIF-1* and *Wnt* pathways, reducing proliferation in *K562* cells and acting as a dual inhibitor. The alkaloid **49a** did not induce activation of the *p53* and *PPARγ* pathways, and this activity was also not detected for alkaloid **49b**.

Another pathway related to the proliferation of abnormal cell is *MAPK/ERK*, which initiates a signaling cascade affecting the endocrine system, regulating cell growth (Li et al., 2023). Thus, the ability of ethanol:water (50%) extract of *T. ignis* to inhibit the *MAPK/ERK*_*1,2*_ pathway in *SW-13* cells was evaluated through an *in vitro* analysis. The hydroalcoholic extract (1 mg mL^-1^) was incubated for 1 h at 37 °C, using saline solution as the control. Antibody dilutions of 1:2,000 for phosphorylated *ERK* (*ERK*_*1*_) and 1:2,500 for total *ERK* (*ERK*_*2*_) were employed (Brown et al., 2001).

The extract showed *MAPK/ERK* pathway activity of 118 ± 16 pmol mg^-1^ protein min^-1^in *SW-13* cells, compared with the control (97 ± 13 pmol mg^-1^ protein min^-1^), indicating the absence of a significant inhibitory effect on this pathway. However, a moderate reduction in *SW-13* cell viability was observed, reaching 73% ± 5% (p < 0.05) (Brown et al., 2001).

Additionally, the hemolytic activity of ethanolic extracts (400 µg extract mL^-1^) from *T. massa* (EETm), *T. charcoti* (EETc), and *T. oxeata* (EETo) was evaluated. by incubating (30 min at 25 °C) the extracts with 100 µL of fresh bovine erythrocyte (pH 7.4) in 96-well microplate, by *in vitro* assay, and was expressed as the half-time of hemolysis (t_50_). The following values were obtained: 0.08 µg, 0.18 µg, and 0.86 µg, respectively. Since hemolysis is associated with cell membrane disruption and consequent toxicity, the cytotoxic effects of EETc and EETo in *CaCo-2* cells may be linked to their membrane-disruptive potential (Berne et al., 2015).

The acetone extract of *T. ignis* was investigated for its ability to inhibit the damage caused by the venoms of *Bothrops jararaca* and *Lachesis muta*, using azocasein (pH 8.8) as a substrate. The venom concentration applied were 34 μg mL^-1^ for *B. jararaca* e 32 μg mL^-1^ for *L. muta*. The activity was determined after 30 min of reaction between the extract and the venom at room temperature. As controls, mixtures of venom with DMSO or venom with saline solution were used, by an *in vitro* assay (Faioli et al., 2013).

Antihemorrhagic activity was evaluated in murine models. The venom dose applied corresponded to 20 μg mL^-1^ (*B. jararaca*) and 10 µg mL^-^1 (*L. muta*), the administration of the sample followed three experimental protocols: (1) prior incubation of the venom with the extract (100 μg mL^-1^) for 30 min, followed by intradermal application; (2) intradermal application of the venom in the mouse, followed by intradermal administration of the extract at the same injection site; and (3) intradermal administration of the venom and intravenous administration of the extract. Two hours after extract administration, the animals were euthanized and the abdominal region (injection site) was collected for evaluation. As negative controls, venom associated with DMSO or venom associated with saline solution were used, by *in vivo* assays (Faioli et al., 2013).

Anti-hemolytic activity was evaluated by the indirect hemolytic test using chicken egg yolk as substrate associated with human erythrocytes. A venom concentration (µg mL^-1^) capable of producing 100% hemolysis, termed the Minimum Indirect Hemolytic Dose (MIHD), was applied. The extract was incubated with the MIHD for 30 min at room temperature. The controls used were similar to those employed in the other assays, by an *in vitro* test (Faioli et al., 2013).

The results showed that the acetone extract (132 μg mL^-1^) inhibited the proteolysis induced by *B. jararaca* venom by 100% and by *L. muta* venom by 70%. Regarding anti-hemorrhagic activity, the acetone extract (220 μg mL^-1^) reduced hemorrhage induced by *B. jararaca* venom by 40% (protocol 1), 45% (protocol 2), and 20% (protocol 3), with a significance level of p < 0.05, while no inhibition was observed against hemorrhage induced by *L. muta* venom. In contrast, the extract (50 μg mL^-1^) inhibited hemolysis induced by *L. muta* venom (25 μg mL^-1^) by 65%, whereas the extract (100 μg mL^-1^) inhibited hemolysis induced by *B. jararaca* venom (50 μg mL^-1^) by less than 20% (Faioli et al., 2013).

### 3.3 Challenge and limitations of available taxonomic, chemistry and biological evidence and prospects for future investigations

Although it is challenging to find comprehensive scientific studies on the genus *Tedania*, the available data reinforce the importance of continuing research on this group of marine sponges. During the preparation of this review, several gaps in knowledge were identified that desserve consideration.

Initially, in was observed that many works did not presente the taxonomic authority of the species, which compromises the standardization of scientific nomenclature. To correct this limitation, the taxoonomic authorities were included by the authors during the review process. For this purpose, information available in the World Register of Marine Species (WoRMS) was consulted, together with general data on geographic distribution and taxonomic aspects obtained from specialized works and reference e-books on Porifera.

Another relevant aspect identified was the low number of metabolites biologically evaluated. Of the total metabolite reported for the genus *Tedania*, only 15.71% were subjected to preclinical assays. In addition, many studies did not establish a direct association between the isolated metabolites and their respective biological activities. In this context, it becomes essential to deepen investigations into the mechanisms of action of these metabolite, as well as their interactions with specific biological targets.

Regarding the experimental approaches used, most studies employed *in vitro* assays, especially microplate and disk diffusion, while a smaller number of works incorporated *in vivo* models, such as zebrafish and mice. Although *in vitro* assays are important tools for initial biological screening, these methods are performed in highly controlled and simplified environments, which may limit the representation of physiological responses observed in living organisms. Thus, the integration of *in vivo* models becomes essential to understand more comprehensively the behavior of metabolites in living organisms.

In addition, *in vitro* methods present specific limitations that must be considered when interpreting the results. In cytotoxicity studies, most works evaluated cell viability through the MTT assay, which acts through a mechanism similar to that of the MTS assay. These methods are based on the ability of the metabolite to reduce cellular metabolismo, which may cause damage without necessarily inducing apoptosis or immediate cell death. Thus, these assays quantify metabolically active cells, which may lead to possible overestimation of cytotoxicity. In contrast, the Sulforhodamide B (SRB) measures the total cellular protein content. In this case, a metabolite may induce cell death without immediately altering this parameter, which may result in possible underestimation of cytotoxicity under certain experimental conditions.

Regarding antimicrobial assays, the most frequently employed method was the determination of the Minimum Innhibitory Concentration (MIC). Although this parameter indicates the ability of the metabolite to inhibit bacterial growth, it does not allow the identification of the mechanism of action nor the evaluation of the kinetics of antimicrobial activity. For a more complete characterization of the activity, it becomes important to incorporate complementary assays, such as the determination of the Minimum Bactericidal Concentration (MBC), which allows distinguishing between bactericidal and bacteriostatic effects. Another relevant assays is the time-kill assays, which enables the analysis of the kinetics of antimicrobial activity and allows a more precise characterization of the behavior of the metabolite against the microorganism.

The investigation of anti-inflammatory activities presentes additional challenges, since the inflammatory response involves a complex network of mediators and signaling pathways. *In vitro* assays generally evaluate only a limited number of signaling pathways, making it difficult to reproduce the complexity observed in real biological systems. In this scenario, the use of *in vivo* models becomes particularly importante, as it allows a more integrated evaluation of the mechanisms involved in the modulation of the inflammatory response.

An example of a comprehensive experimental approach is presented in approach is presented in the study by Jin et al. (2021), in which isolated metabolites were investigated from the chemical isolation process to the evaluation of their cytotoxic activity in diferente cancer cell lines. In addition, the authors explored signaling pathways related to cellular proliferation and survival, as well as mechanisms involved in triggering apoptosis. Also noteworthy is the inclusion of *in vivo* assays to evaluate this latter process, which strengthens the relevance of the results, as it expands the understanding of the biological potential of these metabolites beyond *in vitro* models.

Another factor that may influence experimental results is the physicochemical nature of the extracts and metabolites evaluated. Intensely colored metabolites may interfere with spectrophotometric readings, while poorly soluble metabolites may cause turbidity in the samples, compromising the accuracy and reproducibility of the assays.

From this perspective, several metabolites, fractions, and extracts from the genus *Tedania* deserve deeper investigation in the preclinical context. Thus, the continuation of research with metabolites selected in the initial stages becomes essential, employing pharmacological, pharmacokinetic, and toxicological assays in more complex biological systems. This approach may contribute to identifying the metabolites responsible for the observed activities, elucidating their mechanisms of action, determining the minimum effective doses, and understanding their interactions with the host and wuth biological targets, including possible binding sites to receptors involved in physiopathological processes.

## 4 Patents related to the genus *Tedania*

According to data from the World Intellectual Property Organization (WIPO), which oversees global patent development, the genus *Tedania* has led to the filing of three patent registrations, as summarized in Table 4. Patent No. 115624547 describes the invention of a lead metabolite featuring a novel bioactive structure aimed at the development of drugs for the prevention and treatment of programmed cell necrosis. Patent No. 472297 pertains to the discovery of compound **38** in its pure and natural form. Subsequently, Patent No. 7808142 was granted, covering the tautomeric forms of nucleoside **38**. This metabolite exhibits bioactivity, including muscle relaxant effects that impact the central nervous system (CNS), as well as anti-inflammatory, antiallergic, and hypotensive activities.

**TABLE 4:** Patent registrations of the genus *Tedania*.

Office	Extraction	Publication number	Compound	Title	International classification
China	Broth of the coepiphytic fungus *Aspergillus ochracepetafosterium*, *Tedania* sp	115,624,547	Sesquiterpenoid	Sesquiterpenoids, as well as their preparation method and application	A61K 31/365
					A61K 31/443
					A61K 31/455
					C07D 307/92
					C07D 405/12
					A61P 25/28
					A61P 9/10
					A61P 1/18
					A61P 31/04
					A61P 31/20
					C12P 17/04
					C12P 17/16
					C12R 1/66
					A61P 1/00
Spain	*Tedania* sp	472,297	**38**	Procedure for the Preparation of 1-Methyl-Isoguanosine	A61K
					C07H
					C07H 19/167
					A61K 31/70
					A61K 31/7042
					A61K 31/7052
Holanda	*Tedania* sp	7,808,142	**38**	1-Methyl-Isoguanosine Nucleoside – Isolated from Marine Sponges of the Genus *Tedania* with Muscle Relaxant Activity and Effects on the Central Nervous System (CNS)	C07H 19/052
					C07H 19/16

## 5 Conclusion

This review compiled chemical investigations and biological effects of marine sponge species belonging to the genus *Tedania*, identifying a total of 141 metabolites across various chemical classes over a 48 year period. Data analysis highlights lipids as chemical markers of this genus, with their structures characterized alongside other identified metabolites. Although only 15.5% of the isolated metabolites have undergone biological evaluation, recent studies continue to explore their bioactive potential. This trend reinforces the importance of these metabolites for pharmacological applications, especially with have potential for inhibiting apoptosis, combating microorganisms, and exhibiting anti-inflammatory properties.

Overall, the investigations emphasize the importance of identifying and isolating compounds from marine sponges to enable detailed biological analyses and a better understanding of their mechanisms of action in various therapeutic contexts. This study contributes significantly by providing diverse and integrated information to enhance the understanding of the structural and biological characteristics of metabolites from the genus *Tedania*, aiming to establish correlations between chemical structure and biological activity.

## References

[B1] Acevedo M. S. Puentes C. Carreño K. León J. G. Stupak M. García M. (2013). Antifouling paints based on marine natural products from Colombian Caribbean. Int. Biodeterior. Biodegrad. 83, 97–104. 10.1016/j.ibiod.2013.05.002

[B2] Aiello A. Fattorusso E. Mennat M. Pansini M. (1993). The chemistry of three species of Demospongiae collected from the lagoon of venice: a comparison with some ecological implications. Biochem. Syst. Eco/ogy 21, 655–660. 10.1016/0305-1978(93)90069-4

[B3] Amato A. Esposito R. Federico S. Pozzolini M. Giovine M. Bertolino M. (2023). Marine sponges as promising candidates for integrated aquaculture combining biomass increase and bioremediation: an updated review. Lausanne (Switzerland): Frontiers Media SA, 10. 10.3389/fmars.2023.1234225

[B4] Banias M. P. Uy M. M. (2023). Antiproliferative and pro-apoptotic effects on cancer cell line HCT116 using marine sponge *Tedania* species collected from Kalangahan, Lugait, Misamis Oriental, Philippines. J. Med. Pharm. Allied Sci. 12, 5785–5789. 10.55522/jmpas.V12I3.4971

[B5] Batista D. Costa R. Carvalho A. P. Batista W. R. Rua C. P. J. Oliveira L. (2018). Environmental conditions affect activity and associated microorganisms of marine sponges. Mar. Environ. Res. 142, 59–68. 10.1016/j.marenvres.2018.09.020 30274716

[B6] Berne S. Kalauz M. Lapat M. Savin L. Janussen D. Kersken D. (2015). Screening of the antarctic marine sponges (Porifera) as a source of bioactive compounds. Polar Biol. 39, 947–959. 10.1007/s00300-015-1835-4

[B7] Bocharova E. A. Kopytina N. I. Slynko E. E. (2021). Anti-tumour drugs of marine origin currently at various stages of clinical trials (review). Oles Honchar Dnipro Natl. Univ. 12 (2), 265–280. 10.15421/022136

[B8] Brandão H. N. David J. P. Couto R. D. Nascimento J. A. P. David J. M. (2010). Chemistry and pharmacology of antineoplasic chemoterapeutical derivatives from plants. Quim. Nova 33 (6), 1359–1369. 10.1590/S0100-40422010000600026

[B9] Brown J. W. Kesler C. T. Neary J. T. Fishman L. M. (2001). Effects of marine sponge extracts on mitogen-activated protein kinase (MAPK/ERK 1,2) activity in SW-13 human adrenal carcinoma cells. Toxicon 39, 1835–1839. 10.1016/S0041-0101(01)00138-6 11600145

[B10] Conte A. C. Salles ABCF (2002). A importância do gene p53 na carcinogênese humana. Rev.bras. Hematol. Hemoter 24 (2), 85–89. 10.1590/S1516-

[B11] Costa-Lotufo L. V. Wilke D. V. Jimenez P. C. Epifanio R. d. A. (2009). Marine organisms as a source of new pharmaceuticals: history and perspectives. Quim. Nova 32 (3), 703–716. 10.1590/S0100-40422009000300014

[B12] Costantino V. Fattorusso E. Mangoni A. Perinu C. Cirino G. Gruttola L. (2009). Tedanol: a potent anti-inflammatory ent-pimarane diterpene from the Caribbean Sponge *Tedania ignis* . Bioorg Med. Chem. 17, 7542–7547. 10.1016/j.bmc.2009.09.010 19800802

[B13] Costantino V. Fattorusso E. Mangoni A. Perinu C. Teta R. Panza E. (2012). Tedarenes A and B: structural and stereochemical analysis of two new strained cyclic diarylheptanoids from the marine sponge *Tedania ignis* . J. Org. Chem. 77, 6377–6383. 10.1021/jo300295j 22443364

[B14] Cronan Jr. J. M. Cardellina J. H. II (1994). Cardellina JH. A novel δ-Lactam from the sponge *Tedania ignis* . Nat. Prod. Lett. 5, 85–88. 10.1080/10575639408044039

[B15] De Rosa S. Seizova K. Kamenarska Z. Petrova A. Iodice C. Stefanov K. (2006). Sterol and lipid composition of three adriatic sea sponges. Zeitschrift fur Naturforschung - Sect. C J. Biosci. 61c, 129–134. 10.1515/znc-2006-1-223 16610230

[B16] Dillman R. L. Cardellina J. H. (1991a). An unusual sulfur-containing diketopiperazine from the bermudian sponge *Tedania ignis* . J. Nat. ProdurtJ 54 (4), 1159–1161. 10.1021/np50076a046

[B17] Dillman R. L. Cardellina J. H. (1991b). Aromatic secondary metabolites from the sponge *Tedanla ignis* . J. Nat. Prodwts 54, 1056–1061. 10.1021/np50076a021

[B18] Faioli C. N. Domingos T. F. S. Oliveira E. C. Sanchez E. F. Ribeiro S. Muricy G. (2013). Appraisal of antiophidic potential of marine sponges against Bothrops jararaca and Lachesis muta venom. Toxins 5, 1799–1813. 10.3390/toxins5101799 24141284 PMC3813912

[B19] Guo Z.-J. Liang H.-X. Lian X.-Y. Liao X.-J. Xing X.-W. Xu S.-H. (2024). (+)- and (-)-Tedanine, a pair of new enantiomeric indolone alkaloids from the marine sponge *Tedania* sp. J. Asian Nat. Prod. Res. 26 (3), 328–333. 10.1080/10286020.2023.2244432 37602427

[B20] Hajdu E. Peixinho S. Fernandez J. C. C. (2011). Esponjas marinhas da Bahia: Guia de campo e laboratório, 45. Rio de Janeiro: Museu Nacional, 272–276.

[B21] Hendrayani S.-F. Al-Harbi B. Al-Ansari M. M. Silva G. Aboussekhra A. (2016). The inflammatory/cancer-related IL-6/STAT3/NF-κB positive feedback loop includes AUF1 and maintains the active state of breast myofibroblasts. Oncotarget 7 (27), 41974–41985. 10.18632/oncotarget.9633 27248826 PMC5173109

[B22] Jin T.-Y. Li P.-L. Wang C.-L. Tang X.-L. Cheng M.-M. Zong Y. (2021). Racemic bisindole alkaloids: structure, bioactivity, and computational study. Chin. J. Chem. 39, 39–2598. 10.1002/cjoc.202100255

[B23] Karatepe A. Soylak M. (2014). Sea sponge as a low cost biosorbent for solid phase extraction of some heavy metal ions and determination by flame atomic absorption spectrometry. J. AOAC Int. 97 (6), 1689–1695. 10.5740/jaoacint.12-060 25632444

[B24] Katoh M. Katoh M. (2022). WNT signaling and cancer stemness. Essays Biochem. 66, 319–331. 10.1042/EBC20220016 35837811 PMC9484141

[B25] Kibungu W. C. Clarke A.-M. Fri J. Njom H. A. (2021). Antimicrobial potential and phytochemical screening of *clathria* sp. 1 and *Tedani*a (*Tedania*) stylonychaeta sponge crude extracts obtained from the South East Coast of South Africa. Biomed. Res. Int. 2021, 10. 10.1155/2021/6697944 33728340 PMC7936908

[B26] Knott N. A. Underwood A. J. Chapman M. G. Glasby T. M. (2006). Crescimento da esponja incrustante *Tedania anhelans* (Lieberkuhn) em superfícies verticais e horizontais de recifes subtidais temperados. Mar. Freshw. Res. 57 (1), 95–104. 10.1071/MF05092

[B27] Lhullier C. Moritz M. I. G. Tabalipa E. O. Sardá F. N. Schneider N. F. Z. Moraes M. H. (2020). Biological activities of marine invertebrates extracts from the northeast brazilian coast. Braz. J. Biol. 80 (2), 393–404. 10.1590/1519-6984.213678 31389485

[B28] Li O. Li L. Sheng Y. Ke K. Wu J. Mou Y. (2023). Biological characteristics of pancreatic ductal adenocarcinoma: initiation to malignancy, Cancer Lett. Intracell. Extracelular. Elsevier Ireland Ltd. 574, 216391. 10.1016/j.canlet.2023.216391 37714257

[B29] Marzuki I. Daris L. Nisaa K. Emelda A. (2020). The power of biodegradation and bio-adsorption of bacteria symbiont sponges sea on waste contaminated polycyclic aromatic hydrocarbons and heavy metals. IOP Conf. Ser. Earth Environ. Sci. 584, 012013. 10.1088/1755-1315/584/1/012013

[B30] Monks N. R. Lerner C. Henriques A. T. Farias F. M. Schapoval E. E. S. Suyenaga E. S. (2002). Anticancer, antichemotactic and antimicrobial activities of marine sponges collected off the coast of Santa Catarina, southern Brazil. J. Exp. Mar. Biol. Ecol. 281, 1–12. 10.1016/S0022-0981(02)00380-5

[B31] Montaño M. C. Santafé G. G. Angulo A. Torres O. (2009). Chemical study of the sterolic fractions of marine sponges collected in the caribbean colombian. Rev. Bio. Agro 7 (2), 55–62.

[B32] Nagy M. A. (2011). HIF-1 is the commander of gateways to cancer. J. Cancer Sci. Ther. 3 (2), 35–40. 10.4172/1948-5956.1000054

[B33] Okukado N. (1975). The structure of tedanin, a new carotenoid of *Tedania digitata* (O. Schmidt). Bull. Chemical Society Jpn. 48 (3), 1061–1062. 10.1246/bcsj.48.1061

[B34] Parameswaran P. S. Naik C. G. Hegde V. R. (1997). Secondary metabolites from the sponge *Tedania anhelans*: isolation and characterization of two novel pyrazole acids and other metabolites. J. Nat. Prod. 60 (8), 802–803. 10.1021/np970134z

[B35] Parra L. L. L. (2016). Utilização da desreplicação por HPLC-UV-MS para a descoberta de metabólitos bioativos em invertebrados marinhos. Tese Doutorado. 10.11606/T.75.2016.tde-23032016-093705

[B36] Parra L. L. L. Bertonha A. F. Severo I. R. M. Aguiar A. C. C. Souza G. E. Oliva G. (2018). Isolation, derivative synthesis, and structure-activity relationships of antiparasitic bromopyrrole alkaloids from the marine sponge *Tedania brasiliensis* . J. Nat. Prod. 81, 188–202. 10.1021/acs.jnatprod.7b00876 29297684 PMC5989537

[B37] Patel B. Patel S. P. Balani M. C. (1985). Can a sponge fractionate isotopes? Proc. R. Soc. Lond. B (224), 23–41. 10.1098/rspb.1985.0019 2859596

[B38] Quan D. H. Nagalingam G. Luck I. Proschogo N. Pillalamarri V. Addlagatta A. (2019). Bengamides display potent activity against drug-resistant *Mycobacterium tuberculosis* . Sci. Rep. 9, 14396. 10.1038/s41598-019-50748-2 31591407 PMC6779907

[B39] Quiann R. J. Gregson R. P. Cook A. F. Bartlett R. T. (1980). Isolation and synthesis of i-methylisoguanosine, a potent pharmacologically active constituent from the marine sponge *Tedania digitata* . Tetrahedron Lettrs 21, 567–568. 10.1016/s0040-4039(01)85558-1

[B40] Rod′kina S. A. (2005). Fatty acids from the sponge *Tedania dirhaphis* . Chem. Nat. Compd. 41 (3), 289–292. 10.1007/s10600-005-0131-x

[B41] Saxegaard P. Renstrom B. Litchfield C. Liaaen-Jensen S. Borch A. Tanaka Y. (1981). Identity and stereochemistry of AIIopurpurin and Tedaniaxanthin. Biochem. Syst. Ecol. 9 (4), 325–327. 10.1016/0305-1978(81)90016-8

[B42] Schmidtz F. J. Vanderah D. J. Hollenbeak K. H. Enwall C. E. L. Gopichand Y. SenGupta P. K. (1983). Metabolites from the marine sponge *Tedania ignis*. A new atisanediol and several known diketopiperazines. J. Org. Chem. 48 (22), 3941–3945. 10.1021/jo00170a011

[B43] Schmitz F. J. Gunasekera S. P. Yalamanchili G. Hossain M. B. Helm D. V. (1984). Tedanolide: a potent cytotoxic macrolide from the Caribbean sponge *Tedania ignis* . J. Am. Chem. Soc. 106, 7251–7252. 10.1021/ja00335a069

[B44] Seldes A. M. Gros E. G. Rovirosa J. Vasquez M. L. San-martin A. (1988). Sterols from the marine sponge *Tedania excavata* . Biochem. Syst. Ecol. 16 (5), 495–496. 10.1016/0305-1978(88)90051-8

[B45] Shikov A. N. Flisyuk E. V. Obluchinskaya E. D. Pozharitskaya O. N. (2020). Pharmacokinetics of marine-derived drugs. Mar. Drugs 18, 11–557. 10.3390/md18110557 33182407 PMC7698100

[B46] Souza G. G. O. Castro J. W. G. Nascimento L. L. L. Silva M. I. Leite D. O. D. Santos G. J. G. (2025). Chemical profile, antioxidant and antimicrobial activity of marine sponge species combined with multivariate statistical analyses: *Desmapsamma anchorata, dysidea etheria* and *Echinodictyum dendroides* . Chem. Biodivers. 22 (1), e202402156. 10.1002/cbdv.202402156 39312700

[B47] Stone R. P. Lehnert H. Reiswig H. (2011). A guide to the deep-water sponges of the aleutian island archipelago. NOAA professional paper NMFS, 12. Washington: U.S. Department of Commerce Seattle.

[B48] Tanaka Y. Inoue T. (1988). Occurrence of a new carotenoid 7, 8-Didehydroaaptopurpurin in sea sponge *Tedania digitata* . Nippon. Suisan Gakkaishi 54 (1), 155. 10.2331/suisan.54.155

[B49] Tanaka Y. Katayama T. (1979). Biochemical studies on the carotenoids in porifera the structure of tedaniaxanthin (Part-2). Bull. Jpn. Soc. Sci. Fish. 45 (5), 633–634. 10.2331/suisan.45.633

[B50] Tanaka Y. Katayama T. (1980). Biochemical studies on the carotenoids in porifera the structure of isotedaniaxanthin. Bull. Jpn. Soc. Sci. Fish. 46 (3), 381–383. 10.2331/suisan.46.381

[B51] Tanaka Y. Fujita Y. Katayama T. (1977a). Biochemical studies on the carotenoids in porifera. Identification of the aromatic ketocarotenoid in *Clathria frondifer* and *Tedania digitata* . Bull. Jpn. Soc. Sci. Fish. 43 (6), 767–772. 10.2331/suisan.43.767

[B52] Tanaka Y. Fujita Y. Katayama T. (1977b). Biochemical studies on the carotenoids in porifera. The structure of a new carotenoid, tedaniaxanthin in sea sponge, *Tedania digitata* . Bull. Jpn. Soc. Sci. Fish. 43 (6), 761–765. 10.2331/suisan.43.761

[B53] Tanaka Y. Soejima T. Katayama T. (1978). Biochemical studies of the carotenoids in porifera distribution of the carotenoids in porifer. Bull. Jpn. Soc. Sci. Fish. 44 (11), 1283–1285. 10.2331/suisan.44.1283

[B54] Tanaka Y. Akase S.-I. Yamada S. (2001). Absolute stereochemistry of two carotenoids, clathriaxanthin and isoclathriaxanthin isolated from the marine sponge *Tedania digitata* . Fish. Sci. 67, 378–379. 10.1046/j.1444-2906.2001.00238.x

[B55] Tsurkan D. Wysokowski M. Petrenko I. Voronkina A. Khrunyk Y. Fursov A. (2020). Modern scaffolding strategies based on naturally pre-fabricated 3D biomaterials of poriferan origin. Appl. Phys. A Mater. Sci. Process 126, 382. 10.1007/s00339-020-03564-9

[B56] Uenojo M. Maróstica Jr (2007). MR, Pastore GM Carotenóides: propriedades, aplicações e biotransformação para formação de compostos de aroma. Quim. Nova 30 (3), 616–622. 10.1590/S0100-40422007000300022

[B57] Vidyalakshmi D. Yesudas A. Sivan G. Prakash E. A. Priyaja P. (2024). Heavy metal accumulation analysis using bivalve, sponge, sea urchin, and gastropod species as bioindicators. Mar. Pollut. Bull. 202, 116374. 10.1016/j.marpolbul.2024.116374 38663344

[B58] Wantah E. D. L. Mangindaan R. E. P. Losung F. (2018). Uji aktivitas larvasida dari beberapa ekstrak sponge terhadap larva nyamuk *Aedes aegypti* (Test of larvacide activity from some sponge extracts to *Aedes aegypti* larvae). J. Ilm. Platax 6 (2), 83–88. 10.35800/jip.6.2.2018.20637

[B59] Zhang H. Lai W. Guan Z.-B. Liao X.-J. Zhao B.-X. Xu S.-H. (2020). A new thiodiketopiperzaine from the marine sponge *Tedania* sp. Nat. Prod. Res. 34 (8), 1113–1117. 10.1080/14786419.2018.1550770 30663370

